# Potential for *luxS *related signalling in marine bacteria and production of autoinducer-2 in the genus *Shewanella*

**DOI:** 10.1186/1471-2180-8-13

**Published:** 2008-01-23

**Authors:** Agnes Bodor, Bettina Elxnat, Verena Thiel, Stefan Schulz, Irene Wagner-Döbler

**Affiliations:** 1 Helmholtz-Center for Infection Research, Group Microbial Communication, Division of Cell Biology, Inhoffenstr. 7, 38124 Braunschweig, Germany; 2 Technical University of Braunschweig, Institute of Organic Chemistry, Hagenring 30, 38106 Braunschweig, Germany

## Abstract

**Background:**

The autoinducer-2 (AI-2) group of signalling molecules are produced by both Gram positive and Gram negative bacteria as the by-product of a metabolic transformation carried out by the LuxS enzyme. They are the only non species-specific quorum sensing compounds presently known in bacteria. The *luxS *gene coding for the AI-2 synthase enzyme was found in many important pathogens. Here, we surveyed its occurrence in a collection of 165 marine isolates belonging to abundant marine phyla using conserved degenerated PCR primers and sequencing of selected positive bands to determine if the presence of the *luxS *gene is phylogenetically conserved or dependent on the habitat.

**Results:**

The *luxS *gene was not present in any of the *Alphaproteobacteria *(n = 71) and *Bacteroidetes *strains (n = 29) tested; by contrast, these bacteria harboured the *sahH *gene, coding for an alternative enzyme for the detoxification of *S*-adenosylhomocysteine (SAH) in the activated methyl cycle. Within the *Gammaproteobacteria *(n = 76), *luxS *was found in all *Shewanella, Vibrio *and *Alteromonas *isolates and some *Pseudoalteromonas *and *Halomonas *species, while *sahH *was detected in *Psychrobacter *strains. A number of *Gammaproteobacteria *(n = 27) appeared to have neither the *luxS *nor the *sahH *gene. We then studied the production of AI-2 in the genus *Shewanella *using the *Vibrio harveyi *bioassay. All ten species of *Shewanella *tested produced a pronounced peak of AI-2 towards the end of the exponential growth phase in several media investigated. The maximum of AI-2 activity was different in each *Shewanella *species, ranging from 4% to 46% of the positive control.

**Conclusion:**

The data are consistent with those of fully sequenced bacterial genomes and show that the potential for *lu*xS related signalling is dependent on phylogenetic affiliation rather than ecological niche and is largest in certain groups of *Gammaproteobacteria *in the marine environment. This is the first report on AI-2 production in *Shewanella *species; its signalling role in these organisms remains to be elucidated.

## Background

Cell to cell communication – also called quorum sensing – by self-produced small signalling molecules (autoinducers) is a mechanism by which bacteria coordinate gene expression within their population and in such a way obtain optimum timing and efficiency, for example when infecting a eukaryotic host, degrading polymers, or colonizing surfaces. While most quorum sensing mechanisms studied to date are species specific, Bassler et al. [1] discovered an autoinducer in the marine pathogen *Vibrio harveyi *which is the byproduct of a reaction carried out by a metabolic enzyme and thus has the potential to act as a universal bacterial signal.

Autoinducer-2 (AI-2) refers to a group of compounds resulting from spontaneous cyclisation and hydration of dihydroxypentanedione (DPD), which yields (2S,4S)-2-methyl-2,3,3,4-tetrahydroxytetrahydrofuran (*S*-THMF) and (2R,4S)-2-methyl-2,3,3,4-tetrahydroxytetrahydrofuran (*R*-THMF) in chemical equilibrium. *S*-THMF spontaneously reacts with borate ions to form the active compound detected by *V. harveyi *[2], while *R*-THMF, the other stereoisomere, is unable to complex borate and is sensed by *Salmonella typhimurium *[3] and probably by *Escherichia coli *[4], but not by *V. harveyi*. The receptor proteins for *S*-THMF and *R*-THMF are not homologous, and their three dimensional structures determine the specificity of their interaction with the respective signalling molecules [2,3].

DPD is produced only in the domain Bacteria as a by-product of the activated methyl cycle. The pathway leading to DPD involves a two-step detoxification of *S*-adenosylhomocysteine (SAH), namely by a MTA/SAH nucleosidase (also called Pfs enzyme) and a *S*-ribosylhomocysteinase. The latter enzyme is also known as LuxS enzyme or AI-2 synthase. The products of the reaction carried out by the LuxS enzyme are homocysteine and DPD, which spontaneously forms the AI-2 group of compounds. However, all Eukarya, Archaea, and some phyla within the domain Bacteria employ an alternative one-step detoxification of SAH using only a SAH hydrolase (SahH) enzyme. These bacteria are therefore unable to produce AI-2 [5,6].

The presence of the *luxS *gene (two step detoxification of SAH leading to AI-2) versus the *sahH *gene (one step detoxification) was previously surveyed in the genomes of fully sequenced bacteria [6,7]. Thus, the *luxS *gene was found in some *Gamma*- and *Betaproteobacteria*, all *Epsilonproteobacteria*, none of the *Alphaproteobacteria*, none of the *Actinobacteria*, all *Firmicutes *and some *Spirochaetes*. Since the majority of the fully sequenced bacteria at the time were pathogens, and the number of genomes within the various phylogenetic groups was sometimes very small, it had to remain open if the presence of the *luxS *gene is phylogenetically conserved or if it has been acquired through lateral gene transfer by pathogens.

In the sea, the three most abundant groups of heterotrophic bacteria are members of the *Alphaproteobacteria*, *Gammaproteobacteria*, and *Bacteroidetes *[8-10]. Therefore, here we studied a collection of 165 marine isolates from these three phylogenetic groups [11,12] for the presence of the *luxS *gene using PCR amplification with conserved degenerated primers and sequencing of selected bands. Since the absence of the *luxS *gene could theoretically be due to lack of primer specificity, we confirmed our results by determining the presence of the *sahH *gene, representing the alternative pathway for SAH detoxification. Most bacteria have either the *luxS *or the *sahH *gene in monocopy. There are several exceptions, e.g. *Escherichia blattae *carries both genes, and some bacteria have neither *luxS *nor *sahH *[6,7].

Within the *Gammaproteobacteria*, the *luxS *gene was detected in all *Shewanella *strains consistently. The genus *Shewanella *was first described in 1985 [13] and includes organisms that have been associated with spoilage of proteinacous food (*S. putrefaciens*) as well as opportunistic human pathogens (*S. algae*) [14,15]. During the past two decades, members of the genus *Shewanella *have mostly been studied for their involvement in anaerobic transformations important for biogeochemical cycling of elements. The coupling of growth and energy generation to the dissimilatory reduction of iron and manganese oxide have been demonstrated in *S. oneidensis *MR-1 for the first time in 1988 [16-18]. Although the *luxS *gene has been found in *S. oneidensis *and in several draft genomes of *Shewanella *strains which are currently sequenced, AI-2 production has not yet been investigated in this genus, which comprises both pathogenic and free living species. Therefore, here we determined the ability of nine *Shewanella *type strains and one new marine *Shewanella *isolate to stimulate luminescence of the AI-2 biosensor strain *V. harveyi *BB170 and indeed confirmed the production of AI-2 in all of them for the first time.

## Results

In this study, 148 strains were investigated which have been isolated from various habitats of the North Sea near the island of Helgoland, e.g. the surface of microoalgae (diatoms, dinoflagellates) and macroalgae (laminaria), suspended particles, *in situ *grown biofilms, and the picoplankton [11,19]. In addition, 8 strains from the hypersaline Antarctic Ekho Lake were also included [12]. The marine isolates were identified on the basis of near complete 16S rRNA gene sequences and belonged to 3 different phylogenetic groups: A*lphaproteobacteria *(71 isolates), *Bacteroidetes *(29), and *Gammaproteobacteria *(64). In order to confirm the results which were obtained on the marine *Shewanella *isolates, eight type strains of the genus *Shewanella *and one *Alishewanella fetalis *type strain were also investigated (see Additional file 1 for a complete list of strains tested).

### Identification of the *luxS *gene

All marine isolates were screened with primers LuxS_degfor3 and LuxS_degrev4; for the *Gammaproteobacteria*, the primer set luxS_gamma_for and luxS_gamma_rev (Table 1) was also used. The *luxS *gene was not found in any of the *Alphaproteobacteria *and *Bacteroidetes *strains tested (Additional file 1), consistent with the survey of fully sequenced microbial genomes published previously [6,7].

**Table 1:** Conserved degenerate primers for the *luxS *and *sahH *gene. The luxS gene of *Vibrio harveyi *and the *sahH *gene of *Rhodobacter sphaeroides *served as the reference sequences for primer binding.

**Name**	**Sequence (5'-3')**	**Length**	**Degeneracy**	**Ann. Temp.**	**Start pos.**
LuxS_degfor3	CAT TAT TAG ATA GCT TTA CAD TNG AYC AYA	30 bp	48	48°C	4
LuxS_degrev4	AGC GAG TGC ATC TGA TAA GWN CCR CAY TS	29 bp	64	48°C	410
LuxS_gammafor1	TRG ATA GCT TTA CHG TTG ACC ATA C	25 bp	6	48°C	11
LuxS_gammarev2	TAR AAR CCN GTR CGA CAT CCC AT	23 bp	32	48°C	263
SahH_degfor	GAS GAS ACN ACN ACN GGN GT	20 bp	1024	46°C	562
SahH_degrev	TCV DWR TCR AAR TGD CCR ATR TT	23 bp	1728	46°C	1009
SahH_alphafor	GCH GAR ACV GAR ATG CCS GGB YT	23 bp	432	60°C	64
SahH_alpharev	GAR CCY TTR CCS ACR TYR CCR WA	23 bp	512	60°C	788

In the *Gammaproteobacteria*, the fully sequenced genomes available through NCBI in January 2007 revealed that some representatives use the two step detoxification of SAH, hence contain the *pfs *gene and the *luxS *gene, while others use the one-step detoxification of SAH, thus harbour the *sahH *gene. There are several parasitic and also some free living species which have neither *luxS *nor *sahH*, but only the *pfs *gene. We screened 64 marine *Gammaproteobacteria *for the presence of the *luxS *gene (Additional file 1). It was found in all strains of *Alteromonas*, consistent with the full genome sequence of *Alteromonas hydrophila*. It was also found in seven of 13 strains of *Halomonas *tested; no complete genome sequence is available in this genus yet. In the genus *Pseudoalteromonas*, two genome sequences have been completed, i.e. *P. haloplanktis *and *P. atlantica*. Both species have neither a *luxS *nor a *sahH *homologue, but contain only the *pfs *gene. We tested 28 *Pseudoalteromonas *isolates, and found a PCR band of the expected size in 12 of them. In one strain the band could be confirmed by sequencing to represent a *luxS *gene fragment. Finally, the *luxS *gene was identified in the marine *Shewanella *(three strains) and *Vibrio *(three strains) isolates, as expected from the completed genomes in these genera. In order to determine if the *luxS *gene was consistently present in the *Shewanellaceae *family, nine type strains were investigated (including *A. fetalis*). In all of them the *luxS *gene could be amplified and verified by sequencing.

### Identification of the *sahH *gene

The *sahH *gene is responsible for the *S*-adenosylhomocysteinase enzyme, which is able to detoxify SAH in one step without production of AI-2. In bacteria, usually either *sahH *or *luxS *is present as a single copy. All strains were tested with both *sahH *primers (Table 1). The *sahH *gene was identified in 66 out of 71 *Alphaproteobacteria *and in all 29 *Bacteroidetes *strains (Additional file 1), confirming that the strains from these phyla do not have the ability to produce AI-2 through the LuxS enzyme. Interestingly, the *sahH *gene was also found in some *Gammaproteobacteria*, namely in three strains of *Psychrobacter *sp. (HEL-6, HEL-32 and HEL-44). Two *Psychrobacter *species have been fully sequenced and also have the *sahH *gene (Additional file 1). Full genome sequences of representatives of two additional species of *Gammaproteobacteria *(*Idiomarina loihiensis *and *Saccharophagus degradans*) also revealed the presence of *sahH *(Additional file 1).

### Lack of both the *sahH *and the *luxS *gene

In five of the 31 *Alphaproteobacteria *investigated, and in 27 of the 64 *Gammaproteobacteria *investigated, neither the *luxS *nor the *sahH *gene was detected. Available whole genome sequences of the phylogenetically closest organisms in the NCBI database were checked for comparison, since it can never be excluded that a negative result in PCR is caused by a primer mismatch. Within the *Alphaproteobacteria*, lack of both *luxS *and *sahH *has previously only been seen in the parasitic *Ricksettia *species [7], as well as in the genera *Neoricksettia*, *Wolbachia*, *Anaplasma*, and *Ehrlichia *[6]. In the *Gammaproteobacteria*, the lack of both genes has previously been observed, and SAH recycling presumably occurs through the MTA/SAH nucleoidase alone [7]. Ten of the 27 strains where neither *luxS *nor *sahH *could be identified belonged to the genera *Glaciecola*, *Halomonas *or *Marinobacter*, where genome sequences are not yet available. However, 17 strains belonged into the genus *Pseudoalteromonas*. The fully sequenced genomes of *P. haloplanktis *and *P. atlantica *also have neither *luxS *nor *sahH*, suggesting that this result might not be caused by primer mismatch but is actually correct and indicates SAH detoxification by the Pfs enzyme in these strains. However, confirmation of band identity proved difficult due to the simultaneous amplification of another gene of similar size. It can therefore not be excluded that more *luxS *genes might have been detected using different primers.

### AI-2 production in *Shewanella *and *Alishewanella *species

**Figure 1:**
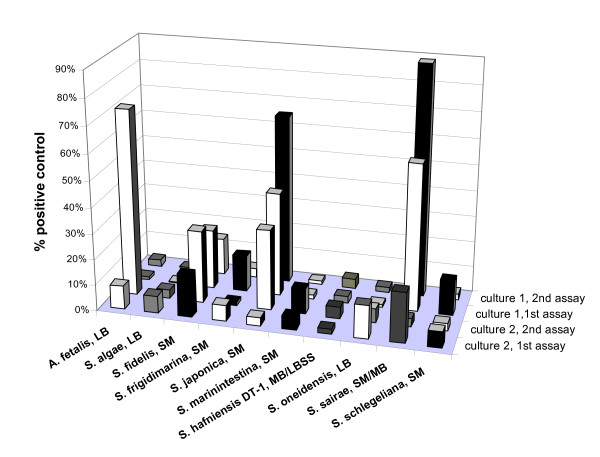
**AI-2 production in different species of *Shewanella***. Peak of AI-2 production during growth in different species of *Shewanella *and *Alishewanella *determined by the *V. harveyi *bioassay. AI-2 production is indicated as percent of positive controls (synthetic DPD or culture supernatant from *V. harveyi *BB152). Conditioned media of the strains were collected from two different cultures throughout growth (culture 1 and culture 2), and tested in the bioassay two times (1^st ^assay and 2^nd ^assay). The filling pattern indicates the type of cultivation medium used: dots, SM medium; vertical bars, LB medium; diagonal bars, MB medium; grid, LBSS medium; S. *Shewanella*; A. *Alishewanella*.

**Figure 2:**
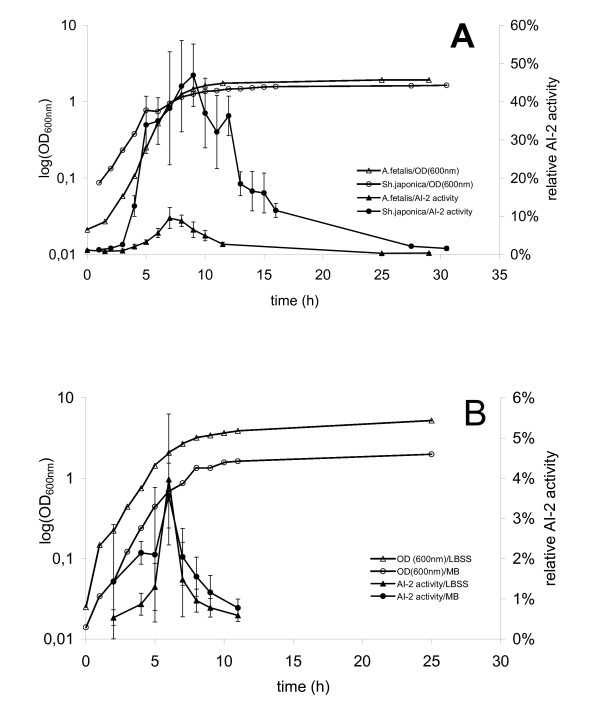
**Effect of growth phase (A) and medium (B) on AI-2 production in representative species of the genus *Shewanella***. (A) Comparison between a *Shewanella *species with low (*A. fetalis*) and high (*S. japonica*) maximum AI-2 level. (B) AI-2 levels during growth for *S. hafniensis *DT-1 cultivated in marine broth (MB) and in Luria Bertani medium with sea salts (LBSS). Fold induction values are given as percentage of the positive control. Standard deviations are given for 4 replicate measurements of the same sample within a bioassay.

Several of the *Gammaproteobacteria *species which did not have the *luxS *gene were tested for AI-2 activity using the *V. harveyi *bioassay. AI-2 could not be detected, confirming the PCR result (data not shown). All *Shewanella *and *Alishewanella *strains contained the *luxS *gene and were tested for AI-2 activity, which was detected in all of them. Table 2 shows the summary of all 73 bioassays conducted; for each species, the maximum A1-2 activity is averaged for the indicated number of bioassays. Average maximum fold induction values were between 4% (e.g. *S. hafniensis*, *S. marinintestina*) and 46% (*S. japonica*). Figure 1 shows a subset of these data; for each species, two different cultures are shown, each of which was tested in two different bioassays independently. Variability between cultures and between replicate bioassays was sometimes very high. Independent of the level of AI-2 activity detected, the same growth phase dependent pattern of production was observed in all investigated strains. Fig. 2A shows this for two representative species, *A. fetalis *with low, and *S. japonica *with high maximum AI-2 activity. In all *Shewanella *strains AI-2 activity reached its maximum towards the end of the exponential growth phase and decreased during stationary phase. In species with high AI-2 activity, this decrease occurred gradually. By contrast, if the AI-2 activity was low, it disappeared rapidly at the beginning of the stationary phase within 2 – 4 hours. At the late stationary phase (after 24 h of growth), no AI-2 activity was detected in any of the strains tested. The same pattern was observed in all cultivation media used. As an example, Fig. 2B shows AI-2 production of *S. hafniensis *DT-1 in two different media. The strain produced similar AI-2 activities at the same growth phase in both of them. *S. oneidensis *was also cultivated anaerobically using iron as an electron acceptor (with 10 and 20 mM ferric citrate, respectively). Under these conditions, growth was very slow and AI-2 activity was not found (data not shown).

**Table 2:** Mean maximum AI-2 activity in *Shewanella *type strains and isolate DT-1. The peak of AI-2 activity (in % of positive controls) during one complete growth curve is averaged for the indicated number of bioassays. Mean ± standard deviation are given. Strain DT-1 was isolated from the surface of the diatom *Thalassiosira sp*. that was picked from a plankton sample (170 μm net) collected near the island of Helgoland in the North Sea on 4^th ^April 2002 (depth 0.5 – 1.5 m).

**Strain**	**Species**	**Peak A1-2 activity [% pos. contr.]**	**no of bio-assays**
CCUG 30811^T^	*Alishewanella fetalis*	24 ± 25^1)^	8
DSM 9167^T^	*Shewanella algae*	9.1 ± 10	13
LMG 20552^T^	*Shewanella fidelis*	18 ± 6	8
LMG 18921^T^	*Shewanella frigidimarina*	7.5 ± 6	6
LMG 19691^T^	*Shewanella japonica*	46.2 ± 33	4
LMG 21403^T^	*Shewanella marinintestina*	3.8 ± 3	10
ATCC 700500^T^	*Shewanella oneidensis *MR-1	9.8 ± 9	10
LMG 21408^T^	*Shewanella sairae*	34 ± 38	5
LMG 21406^T^	*Shewanella schlegeliana*	5.8 ± 5	5
DT-1^2)^	*Shewanella hafniensis*	4.3 ± 1	4

## Discussion

### Phylogenetic distribution of the *sahH *and *luxS *genes

In all *Bacteroidetes *isolates tested here, and in all fully sequenced *Bacteroidetes *strains, the SahH pathway was present, with the exception of *Porphyromonas gingivalis*, which uses the Pfs/LuxS pathway [20,21]. This may indicate that horizontal gene transfer occurred in this species. The main habitat of this bacterium is the oral cavity, where many of the cultivated species use the Pfs/LuxS pathway [22,23] and gene transfer might have a high probability. Several events of gene transfer have been demonstrated for the LuxS enzyme previously [7,24]. A recent report demonstrated AI-2 activity in some *Bacteroides *isolates, although the *luxS *gene could not be identified [25].

Isolates belonging into the *Alphaproteobacteria *had the *sahH *gene consistently and did not contain the *luxS *gene. Therefore, they use the one-step detoxification of SAH and do not have the potential for communication by AI-2. These results confirm previous work based on fully sequenced genomes [5,7].

Within the *Gammaproteobacteria*, the *luxS *gene was found in all strains of the genera *Alteromonas, Shewanella *and *Vibrio *consistent with results from fully sequenced genomes in these genera. It was for the first time found in some strains of the genus *Pseudoalteromonas*. In this genus, whole genome sequencing and PCR amplification indicate that many representatives have neither the *luxS *nor the *sahH *gene and thus use only the Pfs enzyme for removal of SAH.

The same is true for the genus *Halomonas*, where the *luxS *gene was found in some but not all representatives. Species from several other genera of fully sequenced *Gammaproteobacteria *(*Marinobacter*, *Glaciecola*, *Colwellia*) also contained neither the *luxS *nor the *sahH *gene. In addition, in the genus *Psychrobacter *(3 isolates) the *sahH *gene was found consistently, in accordance with fully sequenced genomes in this genus.

The data strongly support the conclusion that the distribution of the *luxS *gene is dependent on the phylogenetic position of an organism rather than on the ecological niche which it has occupied, with rare exceptions which may have been caused by horizontal gene transfer. The complex pattern of presence/absence of *luxS *versus *sahH *in the *Gammaproteobacteria *requires a thorough analysis of the organismal phylogeny and possible gene transfer events in this group, which may not be monophyletic [26,27].

### The genus *Shewanella *and the production of autoinducer-2

The *Shewanella *type strains investigated here were isolated from the sediment of Lake Oneida (*S. oneidensis*) [17], the intestines of marine animals (*S. marinintestina*, *S. sairae*, *S. schlegeliana*)[28], Antarctic sea ice (*S. frigidimarina*) [29], a human fetus (*A. fetalis*) [30], and marine seawater and mussel samples (*S. japonica*) [31]. *S. algae *was isolated from red algae [32] and was differentiated from the first described member of the genus, *S. putrefaciens*, only later[33]. *S. hafniensis *[34] was isolated from fish in the Baltic Sea, and is 98% similar to the marine isolate DT-1 obtained from the surface of a diatom [11].

All *Shewanella*/*Alishewanella *strains were able to induce luminescence in the *V. harveyi *BB170 reporter strain, indicating that they produced the DPD derivative *S*-THMF which can be sensed by *V. harveyi *and which is usually referred to as AI-2. The large differences in the levels of AI-2 seen in the various species of *Shewanella *are partly due to the variability of the bioassay; however compounds inhibitory to *V. harveyi *might also be produced by some strains. For example, stationary phase culture supernatants of *S. algae *and *S. oneidensis *were able to inhibit *V. harveyi *luminescence (data not shown). Alternatively, the highly unstable DPD might be converted to compounds other than AI-2 in *Shewanella*, or the chemical equilibrium between *S*-THMF and *R*-THMF might be shifted towards the *R*-THMF molecule which cannot be detected by *Vibrio*.

### Production of AI-2 during growth

In all *Shewanella *strains, the pattern of AI-2 production during growth was the same. The amount of AI-2 detected in the culture supernatant had a maximum towards the end of the logarithmic growth phase. Subsequently, it decreased and disappeared completely in the late stationary phase. A similar pattern is observed in *Salmonella typhimurium *and *Escherichia coli *[4,35]. In these bacteria, AI-2 triggers its own uptake through induction of an ABC transporter in the late logarithmic growth phase, followed by intracellular degradation. AI-2, or rather, *R*-THMF, is specifically sensed by *Salmonella *through the LsrR protein which bears remote resemblance to ribose transporters. In *Salmonella *and *E. coli*, there is little evidence of cell-cell communication through AI-2. However, nothing is known at the moment about the mechanism responsible for the disappearance of AI-2 from the culture supernatant in *Shewanella *species. A protein with weak homology (27% identity) to the LsrB protein is present in *S. oneidensis*, but most of the other genes in the *lsr *operon are missing.

## Conclusion

In this study, a phylogenetically and ecologically well characterized collection of 165 marine isolates was examined for their potential for communication by AI-2. For this aim, the presence of the *luxS *and *sahH *genes – representing the two pathways for SAH recycling – was determined by PCR with degenerate primers and sequencing. The majority of the isolates tested here belonged to phyla where whole genome sequence analysis had indicated the presence of the *sahH *gene rather than the *luxS *gene (*Alphaproteobacteria, Bacteroidetes*), and this was clearly confirmed in our work. Within the marine *Gammaproteobacteria*, the more complex picture which was already seen in earlier investigations [5-7] was confirmed in this study, with some strains harbouring the *luxS *gene, while the *sahH *gene was present in others, and many strains containing neither of those genes, raising questions regarding the evolution of the *Gammaproteobacteria *and of the Pfs/LuxS and SahH pathways, respectively.

In the second part of our work, we focused on the genus *Shewanella*. The *luxS *gene was consistently found in all marine *Shewanella *isolates and all *Shewanella *type strains tested. The ability to induce luminescence in the *V. harveyi *reporter strain was demonstrated in 10 species of the genus *Shewanella*, both opportunistic pathogens and free living species. The kinetics of AI-2 production was very similar in all *Shewanella *species, showing a peak in the late logarithmic growth phase, and the disappearance of AI-2 from the culture medium during stationary phase. It will be the aim of future studies to elucidate the role of AI-2 in *Shewanella*.

## Methods

### Strains, Media and Cultivation Conditions

A total of 176 strains was investigated, of which 167 had been isolated from marine or hypersaline environments previously [11,12] and 9 were type strains of the genera *Shewanella *and *Alishewanella*. The environmental strains were identified on the basis of near complete 16S rRNA gene sequences as described [11]. A complete list of investigated strains and their phylogenetic affiliation is given in Additional file 1.

*V. harveyi *BB152 ATCC^® ^BAA1119™ and *V. harveyi *BB170 ATCC^® ^BAA-1117™ [1] were used for the bioassay to detect AI-2. *V. harveyi *BB170 carries a transposon insertion in the *luxN *gene, coding for the sensor for AI-1, and is therefore sensitive to AI-2 only. It is used as the sensor strain. *V. harveyi *BB152 carries a transposon insertion in the *luxM *gene responsible for AI-1 synthesis and thus produces AI-2, but not AI-1. Its culture supernatant was used as a positive control [1].

*Shewanella *strains were cultivated under aerobic conditions at room temperature with shaking at 160 rpm in Erlenmeyer flasks, with the exception of *S. oneidensis *(30°C), *A. fetalis *and *S. algae *(37°C), and *S. sairae *(20°C). Media used were Luria broth (LB, Sigma), LBSS (LB with sea salts, composed of LB (Sigma) and 7 g/L sea salts (Sigma)), MB (marine broth, MB 2216, Difco) or SM (*Shewanella *Marine Medium) [36]. SM medium contained 5 g bacto peptone, 1 g yeast extract, 1× salt solution (5× salt solution: 2.58 M NaCl, 0.125 M MgCl_2_, 0.125 M MgSO_4_, 0.1 M KCl) per litre.

*S. algae *was also cultivated under anaerobic conditions without shaking at 30°C in LB medium supplemented with 20 mM Na-*D,L*-lactate (Fluka) and 10 mM or 20 mM ferric citrate (Sigma). Oxygen was removed from the medium by purging with N_2_. The bacterium was inoculated from an aerobic LB plate into anaerobic liquid medium (first transfer). After the third transfer growth was monitored and culture supernatants were collected for AI-2 determination.

*V. harveyi *BB152 and *V. harveyi *BB170 were cultivated in modified autoinducer bioassay (AB) medium [37]. The AB basic solution contained 0.3 M NaCl and 0.05 M MgSO_4_, and was autoclaved at 121°C for 20 min. After cooling down, the following components were added from sterile stocks: 20 ml casamino acids (10%), 20 ml glycerol (50%), 10 ml potassium phosphate buffer (1 M, pH = 7) and 10 ml L-arginine (0.1 M).

Growth was monitored by measuring optical density (OD) at 600 nm in a Ultraspec Plus Spectrophotometer (Pharmacia).

### Design of primers for the *luxS *and *sahH *genes

Degenerate primers were designed targeting highly conserved regions of the *luxS *and *sahH *gene, respectively, based on multiple alignments (ClustalW) of LuxS and SahH amino acid sequences. The alignment of the corresponding nucleic acid sequences was used to decide on a nucleotide in the primer sequence in case of degeneracy of the code.

Since the *luxS *gene is only moderately conserved, the consensus-degenerate hybrid oligonucleotide primer design strategy (CoDeHOP) [38] was chosen for primers LuxS_degfor3 and LuxS_degrev4 (Table 1). According to this approach the primers are composed of two parts: a highly degenerate 5' end and a specific 3' end. The function of the degenerate 5'end is to bind to all possible templates in the first PCR cycles. Therefore, it targeted a region of 3–4 highly conserved amino acids, and covered all possible variations present in the nucleic acid sequence alignment. The role of the specific 3'end is to stabilize the binding of the primers in the later PCR cycles. Its sequence was identical to the most frequent nucleic acid sequence in the alignment and was about 20 bp long. At the time of primer design (July 2004) 24 complete *luxS *sequences of Gram negative bacteria were available. The *luxS *gene is about 500 bp long; and the primer pair LuxS_degfor3 and LuxS_degrev4 amplified ca. 400 bp (Table 1).

In addition, a second, more specific, primer set was designed especially for the *luxS *gene in *Gammaproteobacteria *without using the CoDeHOP approach. The LuxS_gammafor1 primer targeted the same region as LuxS_degfor3. The LuxS_gammarev2 primer targeted the highly conserved mGC*TG*y region in the middle of the *luxS *gene. A fragment of about 250 bp was amplified with this primer set.

The *sahH *gene is highly conserved and much longer than the *luxS *gene (about 1400 bp). Therefore, longer conserved amino acid sequences could be identified and it was not necessary to use the CoDeHOP strategy. 60 bacterial *sahH *sequences were used for the alignment and a universal primer set (SahH_degfor and SahH_degrev; see Table 1) was designed which worked from 40°C till 60°C and amplified about 470 bp.

In some *Alphaproteobacteria*, these primers did not amplify the *sahH *gene. Therefore primers specific for this group were designed (Table 1). Primer SahH_alphafor targeted the AETEMPGL region that is completely identical in all sequenced *sahH *genes in *Alphaproteobacteria*. The primer SahH_alpharev targeted the G*G*VGKGS conserved region around the middle of the gene which is part of the NAD binding domain reported previously [39]. This primer set amplified about 700 bp and was able to amplify *sahH *genes in additional *Alphaproteobacteria *at an annealing temperature of 60°C.

### Polymerase Chain Reaction (PCR) and Sequencing

DNA was isolated using the Nucleospin Tissue kit (Macherey & Nagel). The PCR primers are listed in Table 1. The PCR reaction mix contained: 1× PCR buffer (Qiagen); 3 mM MgCl_2 _(Qiagen); 1.25 U *Taq *polymerase (Qiagen); 1 μM forward and reverse primer each (Gibco); 200 μM dNTPs (Fermentas); 1 μl DNA and MilliQ water (Fluka) up to an end volume of 50 μl. The PCR protocol was as follows: Predenaturation at 94°C for 1 min, followed by 30 cycles of denaturation at 94°C for 10 sec, annealing for 30 sec at the temperatures given in Table 1, and elongation at 68°C for 40 sec (*luxS *gene) resp. 50 sec (*sahH *gene); finally postelongation was carried out at 68°C for 1 min 30 sec. The annealing temperatures listed in Table 1 were determined by PCR with a temperature gradient from 40 to 60°C. All of the PCRs were done in an Eppendorf Mastercycler.

The PCR products were visualised on 1.5% agarose gels (agarose NEEO ultra-quality, Roth). Bands that were chosen for sequencing were excised from the gel and purified using the Qiagen Gel Extraction Kit. Excised bands were sequenced using the BigDye Terminator v3.1 Cycle Sequencing Kit on an ABI 3730 × l DNA analyser.

### Determination of AI-2 using the *Vibrio harveyi *bioassay

AI-2 activity was always determined throughout the complete growth curve for at least 24 h. Samples were taken every 30 – 60 min until stationary phase was reached, then larger sampling intervals were used. Sterile culture supernatants were prepared from 1 ml of culture fluid by centrifugation (13.000 rpm, 2 min) and filtration (0.22 μm) and were stored at -20°C until use. The assay was conducted in white micro titre plates (Nunc) which were prepared before the assay by pipetting 20 μl of samples, reference and control in at least four replicas into the wells. Plates were stored at -20°C until use. DPD was synthesized as described [40]. The concentration of DPD (M = 132 g/mol) was 0.7 mg/ml, e.g. ca. 5.3 mM. This solution was diluted 1:1000 in AB medium and served as a positive control. In some bioassays culture supernatant from *V. harveyi *BB152 served as a positive control.

The assay described by Bassler [41] was slightly modified [42]. Briefly, the sensor strain *V. harveyi *BB170 was inoculated from a freshly prepared AB plate with a well luminescent culture into 20 ml AB liquid medium in an Erlenmeyer flask with baffles and incubated (160 rpm, 30°C) until it reached an OD_600nm _= 1.0 and showed strong luminescence in the dark room. At this growth stage the sensor strain was diluted 1:5000 in fresh AB medium. 180 μl of the diluted culture were immediately added to the previously prepared, warmed-up micro titre plates with the samples. Plates were incubated at 30°C with shaking at 600 rpm on a Heidolph Titramax -1000 micro titre plate shaker and luminescence was measured every hour for up to 8 hours in a Wallac Victor 1420 Multilabel Counter (Perkin Elmer). AI-2 activity was calculated as the fold change between luminescence of the sample and luminescence of the reference. The reference for the positive control was AB medium, and the reference for the test samples was the growth medium in which the respective test strain had been cultivated. The percentage of AI-2 activity was calculated as the fold induction of test sample divided by that of the positive control.

## Authors' contributions

AB performed most of the experiments, analysed the data, provided figures and tables and helped with writing the manuscript, BE did some experiments and analysed data, VT synthesized AI-2, SS improved the method for AI-2 synthesis, and IW-D designed the study and wrote the paper. All authors have read and approved the final version of the manuscript.

## Supplementary Material

Additional file 1 Additional File 1. Complete list of strains tested in this work and results of PCR screening and sequencing with respect to the presence of the *luxS *and *sahH *gene. Phylogenetic affiliation was determined by BLAST search of 16S rRNA sequences (about 1200 nt length in the *Alphaproteobacteria*, and about 600 nt starting with position 27 (*E. coli *numbering) in all other strains) and is shown as % similarity with the nearest described type strain. The presence/absence of the *luxS *gene and the *sahH *gene were determined by presence/absence of a band of the expected size by PCR; bands confirmed by sequencing are indicated and shown in bold face. Within the *Gammaproteobacteria*, several fully sequenced species are also listed (shaded in yellow) and the presence of *luxS *resp. *sahH *is indicated. Click here for file

## References

[B1] Bassler BL, Wright M, Showalter RE, Silverman MR (1993). Intercellular signalling in Vibrio harveyi: sequence and function of genes regulating expression of luminescence. Mol Microbiol.

[B2] Chen X, Schauder S, Potier N, Van Dorsselaer A, Pelczer I, Bassler BL, Hughson FM (2002). Structural identification of a bacterial quorum-sensing signal containing boron. Nature.

[B3] Miller ST, Xavier KB, Campagna SR, Taga ME, Semmelhack MF, Bassler BL, Hughson FM (2004). Salmonella typhimurium recognizes a chemically distinct form of the bacterial quorum-sensing signal AI-2. Mol Cell.

[B4] Xavier KB, Bassler BL (2005). Regulation of uptake and processing of the quorum-sensing autoinducer AI-2 in Escherichia coli. J Bacteriol.

[B5] Winzer K, Hardie KR, Burgess N, Doherty N, Kirke D, Holden MT, Linforth R, Cornell KA, Taylor AJ, Hill PJ, Williams P (2002). LuxS: its role in central metabolism and the in vitro synthesis of 4-hydroxy-5-methyl-3(2H)-furanone. Microbiology.

[B6] Vendeville A, Winzer K, Heurlier K, Tang CM, Hardie KR (2005). Making 'sense' of metabolism: autoinducer-2, LuxS and pathogenic bacteria. Nat Rev Microbiol.

[B7] Sun J, Daniel R, Wagner-Dobler I, Zeng AP (2004). Is autoinducer-2 a universal signal for interspecies communication: a comparative genomic and phylogenetic analysis of the synthesis and signal transduction pathways. BMC Evol Biol.

[B8] Glockner FO, Fuchs BM, Amann R (1999). Bacterioplankton compositions of lakes and oceans: a first comparison based on fluorescence in situ hybridization. Appl Environ Microbiol.

[B9] Pommier T, Canback B, Riemann L, Bostrom KH, Simu K, Lundberg P, Tunlid A, Hagstrom A (2007). Global patterns of diversity and community structure in marine bacterioplankton. Mol Ecol.

[B10] Fuchs BM, Spring S, Teeling H, Quast C, Wulf J, Schattenhofer M, Yan S, Ferriera S, Johnson J, Glöckner FO, Amann R (2007). Characterization of a marine gammaproteobacterium capable of aerobic anoxygenic photosynthesis. Proc Natl Acad Sci U S A.

[B11] Allgaier M, Uphoff H, Felske A, Wagner-Dobler I (2003). Aerobic anoxygenic photosynthesis in Roseobacter clade bacteria from diverse marine habitats. Appl Environ Microbiol.

[B12] Labrenz M, Collins MD, Lawson PA, Tindall BJ, Schumann P, Hirsch P (1999). Roseovarius tolerans gen. nov., sp. nov., a budding bacterium with variable bacteriochlorophyll a production from hypersaline Ekho Lake. Int J Syst Bacteriol.

[B13] MacDonell MT, Colwell RR (1985). Phylogeny of the Vibrionaceae, and recommendation for two new genera, Listonella and Shewanella. Syst Appl Microbiol.

[B14] Holt HM, Gahrn-Hansen B, Bruun B (2005). Shewanella algae and Shewanella putrefaciens: clinical and microbiological characteristics. Clin Microbiol Infect.

[B15] Liu MC, Gau SJ, Wu HC (2006). Acute exudative tonsillitis caused by Shewanella algae in a healthy child. Scand J Infect Dis.

[B16] Nealson KH, Saffarini D (1994). Iron and manganese in anaerobic respiration: environmental significance, physiology, and regulation. Annu Rev Microbiol.

[B17] Heidelberg JF, Paulsen IT, Nelson KE, Gaidos EJ, Nelson WC, Read TD, Eisen JA, Seshadri R, Ward N, Methe B, Clayton RA, Meyer T, Tsapin A, Scott J, Beanan M, Brinkac L, Daugherty S, DeBoy RT, Dodson RJ, Durkin AS, Haft DH, Kolonay JF, Madupu R, Peterson JD, Umayam LA, White O, Wolf AM, Vamathevan J, Weidman J, Impraim M, Lee K, Berry K, Lee C, Mueller J, Khouri H, Gill J, Utterback TR, McDonald LA, Feldblyum TV, Smith HO, Venter JC, Nealson KH, Fraser CM (2002). Genome sequence of the dissimilatory metal ion-reducing bacterium Shewanella oneidensis. Nat Biotechnol.

[B18] Venkateswaran K, Moser DP, Dollhopf ME, Lies DP, Saffarini DA, MacGregor BJ, Ringelberg DB, White DC, Nishijima M, Sano H, Burghardt J, Stackebrandt E, Nealson KH (1999). Polyphasic taxonomy of the genus Shewanella and description of Shewanella oneidensis sp. nov. Int J Syst Bacteriol.

[B19] Uphoff HU, Felske A, Fehr W, Wagner-Dobler I (2001). The microbial diversity in picoplankton enrichment cultures: a molecular screening of marine isolates. FEMS Microbiol Ecol.

[B20] Chung WO, Park Y, Lamont RJ, McNab R, Barbieri B, Demuth DR (2001). Signaling system in Porphyromonas gingivalis based on a LuxS protein. J Bacteriol.

[B21] James CE, Hasegawa Y, Park Y, Yeung V, Tribble GD, Kuboniwa M, Demuth DR, Lamont RJ (2006). LuxS involvement in the regulation of genes coding for hemin and iron acquisition systems in Porphyromonas gingivalis. Infect Immun.

[B22] Frias J, Olle E, Alsina M (2001). Periodontal pathogens produce quorum sensing signal molecules. Infect Immun.

[B23] Kolenbrander PE, Andersen RN, Blehert DS, Egland PG, Foster JS, Palmer RJ (2002). Communication among oral bacteria. Microbiol Mol Biol Rev.

[B24] Lerat E, Moran NA (2004). The evolutionary history of quorum-sensing systems in bacteria. Mol Biol Evol.

[B25] Antunes LC, Queiroz FL, Oliveira FE, Rodrigues MK, Eliane Santos AK, Maria Cavalcanti Pilotto DR, Candida de Souza FM (2005). Bacteroides species produce Vibrio harveyi autoinducer 2-related molecules. Anaerobe.

[B26] Lerat E, Daubin V, Moran NA (2003). From gene trees to organismal phylogeny in prokaryotes: the case of the gamma-Proteobacteria. PLoS Biol.

[B27] Brown JR, Volker C (2004). Phylogeny of gamma-proteobacteria: resolution of one branch of the universal tree?. Bioessays.

[B28] Satomi M, Oikawa H, Yano Y (2003). Shewanella marinintestina sp. nov., Shewanella schlegeliana sp. nov. and Shewanella sairae sp. nov., novel eicosapentaenoic-acid-producing marine bacteria isolated from sea-animal intestines. Int J Syst Evol Microbiol.

[B29] Bowman JP, McCammon SA, Nichols DS, Skerratt JH, Rea SM, Nichols PD, McMeekin TA (1997). Shewanella gelidimarina sp. nov. and Shewanella frigidimarina sp. nov., novel Antarctic species with the ability to produce eicosapentaenoic acid (20:5 omega 3) and grow anaerobically by dissimilatory Fe(III) reduction. Int J Syst Bacteriol.

[B30] Vogel BF, Venkateswaran K, Christensen H, Falsen E, Christiansen G, Gram L (2000). Polyphasic taxonomic approach in the description of Alishewanella fetalis gen. nov., sp. nov., isolated from a human foetus. Int J Syst Evol Microbiol.

[B31] Ivanova EP, Sawabe T, Gorshkova NM, Svetashev VI, Mikhailov VV, Nicolau DV, Christen R (2001). Shewanella japonica sp. nov. Int J Syst Evol Microbiol.

[B32] Simidu U, Kita-Tsukamoto K, Yasumoto T, Yotsu M (1990). Taxonomy of four marine bacterial strains that produce tetrodotoxin. Int J Syst Bacteriol.

[B33] Vogel BF, Jorgensen K, Christensen H, Olsen JE, Gram L (1997). Differentiation of Shewanella putrefaciens and Shewanella alga on the basis of whole-cell protein profiles, ribotyping, phenotypic characterization, and 16S rRNA gene sequence analysis. Appl Environ Microbiol.

[B34] Satomi M, Vogel BF, Gram L, Venkateswaran K (2006). Shewanella hafniensis sp. nov. and Shewanella morhuae sp. nov., isolated from marine fish of the Baltic Sea. Int J Syst Evol Microbiol.

[B35] Taga ME, Semmelhack JL, Bassler BL (2001). The LuxS-dependent autoinducer AI-2 controls the expression of an ABC transporter that functions in AI-2 uptake in Salmonella typhimurium. Mol Microbiol.

[B36] Makemson JC, Fulayfil NR, Landry W, Van Ert LM, Wimpee CF, Widder EA, Case JF (1997). Shewanella woodyi sp. nov., an exclusively respiratory luminous bacterium isolated from the Alboran Sea. Int J Syst Bacteriol.

[B37] Greenberg EP, Hastings JW, Ulitzur S (1979). Induction of Luciferase Synthesis in Beneckea harveyi by Other Marine Bacteria. Arch Microbiol.

[B38] Rose TM, Henikoff JG, Henikoff S (2003). CODEHOP (COnsensus-DEgenerate Hybrid Oligonucleotide Primer) PCR primer design. Nucleic Acids Res.

[B39] Sganga MW, Aksamit RR, Cantoni GL, Bauer CE (1992). Mutational and nucleotide sequence analysis of S-adenosyl-L-homocysteine hydrolase from Rhodobacter capsulatus. Proc Natl Acad Sci U S A.

[B40] De Keersmaecker SC, Varszegi C, van Boxel N, Habel LW, Metzger K, Daniels R, Marchal K, De Vos D, Vanderleyden J (2005). Chemical synthesis of (S)-4,5-dihydroxy-2,3-pentanedione, a bacterial signal molecule precursor, and validation of its activity in Salmonella typhimurium. J Biol Chem.

[B41] Bassler BL, Greenberg EP, Stevens AM (1997). Cross-species induction of luminescence in the quorum-sensing bacterium Vibrio harveyi. J Bacteriol.

[B42] Vilchez R, Lemme A, Thiel V, Schulz S, Sztajer H, Wagner-Dobler I (2007). Analysing traces of autoinducer-2 requires standardization of the Vibrio harveyi bioassay. Anal Bioanal Chem.

